# Women’s knowledge towards neonatal danger signs and its associated factors in Ethiopia: a systematic review and meta-analysis

**DOI:** 10.1186/s12887-020-02098-6

**Published:** 2020-05-14

**Authors:** Asmamaw Demis, Getnet Gedefaw, Adam Wondmieneh, Addisu Getie, Birhan Alemnew

**Affiliations:** 1 Department of Nursing, College of Health Sciences, Woldia University, P.O. Box:400, Woldia, Ethiopia; 2 Department of Midwifery, College of Health Sciences, Woldia University, P.O.Box:400, Woldia, Ethiopia; 3 Department of Medical Laboratory Sciences, College of Health Sciences, Woldia University, P.O.Box:400, Woldia, Ethiopia

**Keywords:** Danger signs, Newborn, Systematic review, Ethiopia

## Abstract

**Introduction:**

Reducing neonatal mortality is an essential part of the third Sustainable Development Goal, to end preventable child deaths. Neonatal danger signs are the most common cause of neonatal mortality and morbidity. In Ethiopia, most babies are born at home or are discharged from the health institutions in the first 24 h, as a result enhancing women’s knowledge towards neonatal danger signs and its complication might reduce neonatal morbidity and mortality. Therefore, this systematic review and meta-analysis aimed to assess the women knowledge towards neonatal danger signs in Ethiopia.

**Method:**

MEDLINE/PubMed, Scopus, Hinari, Google scholar, web of science electronic databases and grey literature from repository were searched for all the available studies. Fourteen cross sectional studies were included in this systematic review and meta-analysis. Subgroup analysis was conducted for the evidence of heterogeneity. Cochrane I^2^ statistics were used to check the heterogeneity of the studies. Egger test with funnel plot were used to investigate publication bias.

**Result:**

Fourteen cross-sectional studies with a total of 6617 study participants were included for this study. The overall pooled prevalence of women’s knowledge towards neonatal danger sign was 40.7% (95%CI, 25.72, 55.67). Having higher educational status of the women (AOR = 3.86, 95%CI: 2.3–6.5), having higher educational status of the husband (AOR = 4.57, 95%CI: 3.29–6.35), access to mass media (AOR = 1.69, 95%CI: 1.17–2.23), having antenatal care visits (AOR = 2.63, 95%CI: 1.13–4.67), having postnatal care follow up (AOR = 2.55, 95%CI; 1.72–3.79) and giving birth at health institutions (AOR = 2.51, 95%CI:1.68–3.74) were factors associated with knowledge of the women towards danger sign of the neonate.

**Conclusion:**

In this systematic review and meta-analysis the pooled prevalence of maternal knowledge towards neonatal danger sign was low. Educational status of the mother, educational status of the husband, access to mass media, antenatal care follow-up, postnatal care follow-up and place of delivery were factors associated with knowledge of the mother towards danger sign of the newborn. Promoting antenatal care, postnatal care follow-up and community-based health information dissemination about neonatal danger signs should be strengthened.

**Systematic review registration:**

PROSPERO CRD42019132179.

## Introduction

Neonates are the most vulnerable age group of the human population. They aren’t small adults, therefore they need to be regarded with special nursery and special care [1]. Neonatal danger signs are common and easy signs to recognize, associated with a potentially severe problem that can be easily identified by non-clinical personnel including the mother and other family members [2].

Women’s knowledge of neonatal danger sign is crucial to influence their decisions to seek immediate health care for their sick neonate who contributes a lot in reducing neonatal morbidity, mortality and related to disease presented with danger signs [3, 4]. Globally, 2.5 million neonates died during the neonatal period with approximately 7000 newborn deaths every day with about one third dying on the day of birth and near to three quarters dying within the first week of life accounting to 47% of all child deaths under the age of 5-years [5].

Worldwide, every year about four million babies die in the neonatal period (the first month of life) which accounts for 38% of all deaths in children younger than age 5 years. Despite, the number of neonatal deaths declined from 5 million in 1990 to 2.5 million in 2018 globally, decreasing neonatal mortality in Sub-Saharan Africa and southern Asia is difficult to avert the significant burden of neonatal mortality [5–7].

Neonatal mortality rates were varying widely across the world, however the highest neonatal death occurred in 2018 Sub-Saharan Africa and Central and Southern Asia which accounts 28 and 25 deaths per 1000 live births respectively. If each country achieves the SDG neonatal mortality target of 12 deaths per 1000 live births or fewer by 2030, it was projected that 22.7 million cumulative neonatal deaths by 2030 [7, 8]. Neonatal deaths in sub-Saharan Africa or in Southern Asia are more than 10 times likely to die in the first month than a child born in developed countries. Majority of neonatal mortality in low and middle income countries happened at home in settings where a few women’s and family members recognize signs of newborn illness and nearly all neonates are not taken to health facilities when they were sick [7–9].

Nearly 75% of neonatal deaths could be avoided with simple low-cost effective methods if the neonate illness early recognized came to the health facility and neonate receive timely quality neonatal care. Even though in settings with well-functioning midwife programmes the provision of midwife-led continuity of care (MLCC) can reduce preterm births by up to 24%, in low and middle income countries is significantly challenging [10]. In Ethiopia, neonatal mortality declined more slowly than mortality among children aged 0–4 years. As a result, the share of neonatal deaths among all under-five deaths increased from 29% 1000 live births in 2016 Ethiopian demographic health survey (EDHS) to 30% 1000 live births in 2019 Ethiopian mini demographic health survey (EMDHS) [11, 12]. Lack of quality care at birth or skilled care and treatment immediately after birth and in the first days of life were the associated factors for neonatal morbidity and mortality. Preterm birth (prematurity), labor and delivery related complications (birth asphyxia, meconium stained amniotic fluid, hypothermia, hyperthermia, respiratory distress syndrome, or lack of breathing at birth), infections and birth defects were the commonest cause of neonatal deaths [7].

Improving the quality of maternal and newborn care from pregnancy to postnatal period, encouraging the quality of care given during the first week of neonatal life, and expanding quality services for small and sick newborns were the recommended strategy to prevent neonatal danger signs and its complications [4, 7]. In Ethiopia, there were several studies about women’s knowledge of neonatal danger signs and its associated factors. Most of the available studies are cross-sectional in design and conducted in limited areas which didn’t address all regions of the country; hence, we are unable to indicate more accurately women’s knowledge of neonatal danger signs at the national level. As a result, this systematic review would help policymakers and health managers and planners to make evidence-based decisions that have taken into account all the available information, as well as providing an indication as to the quality of the results. Therefore, this systematic review is designed to identify the level of women’s knowledge of neonate/newborn danger signs to present accurate information that could be used in policy formulation and practice evidence-based decision-making.

## Methods

### Study design and setting

Ethiopia is one of low-income countries located in Eastern Africa with a total fertility rate of 4.6. This systematic review and meta-analysis were conducted to estimate the pooled prevalence of women’s knowledge towards neonatal danger signs and its associated factors in Ethiopia.

### Search strategies

Studies were searched from online databases including MEDLINE/PubMed, Scopus, Web of Science, Maternity and Infant Care and Wiley Online Library. Additionally, bibliographies of identified articles and grey literature, including Google scholar, MEDNAR, and World Wide Science were searched. Moreover, missing data were handled by contacting corresponding authors. Search terms were formulating using PICO guidelines through the online databases and comprehensive search strategy had been developed using different Boolean operators.

The following search terms were used: Knowledge OR Awareness OR Understanding AND “Neonatal danger signs” OR “newborn danger signs” OR “Warning signs of newborn” OR “Neonatal warning signs” OR “Unable to breastfeeding” OR “Convulsion” OR “Lethargy” OR “Difficulty in breathing” OR “Jaundice” OR “Hypothermia” OR “Hyperthermia” OR “Pus discharge” OR “Repeated Vomiting” AND “Mother’s” OR “Women” AND “Associated factors” AND Ethiopia and related terms. Systematic review with narrative synthesis was used to summarize the findings of articles in Ethiopia.

### Eligibility criteria

#### Inclusion criteria

##### Population

Antenatal and postnatal women were included.

##### Study design

Observational studies (cross-sectional, case-control, and retrospective and prospective cohort studies and national survey and surveillance reports) were included.

##### Study area

only studies conducted in Ethiopia without time limiting and reported the magnitude or at least one least adjusted associated factor of knowledge of neonatal danger signs among mother was included.

##### Publication status and language

Both published and unpublished reported articles in English language only were considered.

##### Searching date

Studies published till September 2/2019 were included.

#### Exclusion criteria

Citations without abstracts and/or full-text, commentaries, anonymous reports, letters, editorials and articles not reporting the outcome of the study were excluded after reviewing the full texts.

#### Outcome measurements

This systematic review and meta-analysis had two essential outcomes. These were:

##### Primary outcome

The level of knowledge of women’s towards neonatal danger signs.

##### Secondary outcome

Factors affecting knowledge of women’s towards neonatal danger signs which were measured by higher level of maternal educational status (yes/no), higher educational level of the husband (yes/no), access to mass media (yes/no), attending antenatal care visits (yes/no), attending postnatal care follow up (yes/no), place of delivery (health facility/home) were the main contributing factors for neonatal danger signs.

### Data extraction

First, all studies obtained from all databases were exported to Endnote version X8 software to remove duplicates. Then after, all studies were exported to Microsoft Excel spreadsheet. Two authors (AD and GG) independently extracted all the important data using a standardized data extraction form which was adapted from the JBI data extraction format. Substantial agreement between reviewers i.e. Cohen’s kappa coefficient > 0.60 was accepted and resolved through discussion and consensus. For the first outcome (prevalence) the data extraction format included (primary author, year of publication, regions, study area, sample size, and prevalence with 95%CI). For the second outcome (associated factors) data were extracted with 2 by 2 table format and then the log odds ratio for each factor was calculated.

### Quality assessment

Two authors (AD&GG) independently assessed the quality of each studies using Newcastle-Ottawa-scale (NOS) for cross-sectional studies [13]. All Articles underwent systematic review and meta-analysis was cross-sectional studies. The methodological quality, comparability and the outcome and statistical analysis of the study were the three major assessment tools used to declare the quality of the study. Lastly, studies scored a scale of ≥7 out of 10 was considered as achieving high quality. During quality appraisal of the articles, any discrepancies between the two authors were resolved by taking the second group authors (AW, AG and BA). All of the studies were included based on the Newcastle –Ottawa Scale quality assessment criteria.

### Data processing and analysis

Random effect model was applied to estimate the pooled prevalence of having good knowledge of neonatal danger signs among postnatal women. After extraction of the articles in Microsoft Excel spreadsheet format, the analysis was carried out using STATA version 11 statistical software. Cochrane Q-test and *I*^2^statistics were computed to assess heterogeneity among studies [14]. After computing the statistics, results showed there is significant heterogeneity among studies (*I*^2^ = 99.6%, *p* < 0.001). To estimate the overall prevalence of having good knowledge of the women, via back-transform of the weighted mean of the transformed proportions arcsine variance weights and Dersimonian-Laird weights for fixed-effects model and random effect model respectively [15]. Publication bias was assessed using egger’s test. Subgroup analysis was done based on study setting (facility vs community based), sample size and women’s spontaneous response to minimize the random variations between the point estimates of the primary study. Forest plot format was used to present the pooled point prevalence with 95%Cl. For associations, a log odds ratio was used to decide the association between associated factors and having good knowledge among mothers towards neonatal danger signs in the included studies.

## Results

**Fig. 1:**
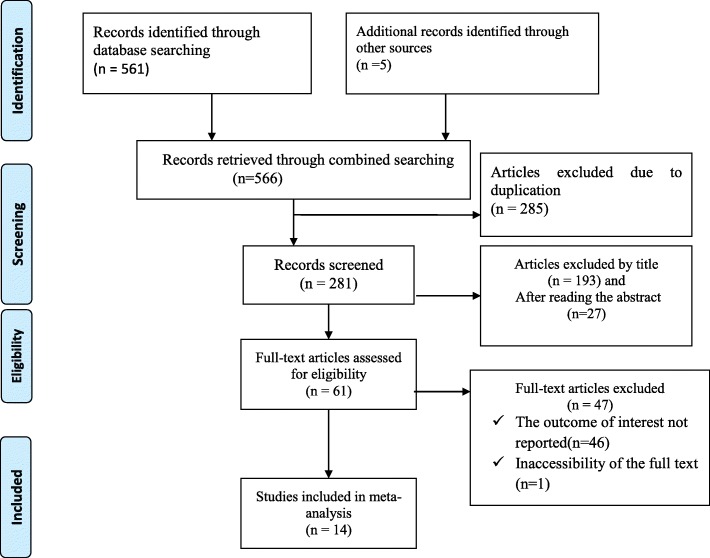
Flow chart of selection for systematic review and meta-analysis of women’s knowledge towards neonatal danger signs and its associated factors in Ethiopia

n the first step of our search, 566 articles were retrieved regarding the prevalence and associated factors of knowledge among postnatal women at MEDLINE/PubMed, Google Scholar, Web of Science and other sources described previously. Of 566 articles, 285 articles were excluded due to duplication. From the remaining 281 articles, 220 articles were excluded after review of their titles and abstracts due to as non-relevant to this review. Therefore, 61full-text articles were accessed and assessed for eligibility based on the pre-set criteria, which resulted in the further exclusion of 47articles primarily due to reason. As a result, 14 studies met the eligibility criteria and were included in the final meta-analysis (Fig. 1).

### Characteristics of original studies

Among 14 articles which were published in Ethiopia until 2019, 6617 study participants were involved to determine the pooled prevalence of newborn danger signs among mothers. Regarding the study design, all the studies are cross-sectional. The sample size of the studies was ranged from 197 to 845. Three of the studies were from Amhara region [16–18], three from SNNPR [19–21], three from Oromia region [22–24], three from Tigray region [25–27], one from Addis Ababa [28]**,** and one from Harar regional state [29] (Table 1).

**Table 1:** Study characteristics included in the systematic review and meta-analysis on knowledge of neonatal danger signs and associated factors among women in Ethiopia

Authors	Region	Study area	Publication year	Spontaneous response	Study setting	Study design	Sample size	Prevalence
Nigatu et al, (2015) [18]	Amhara	Gondar	2015	At least three	Community	Cross-sectional	603	18.2
Yadeta TA, (2018) [23]	Oromia	East Hararge	2018	At least four	Community	Cross-sectional	757	9.4
Mersha A et al., (2017) [20]	SSNPR	Chencha	2017	At least three	Community	Cross-sectional	630	50.3
Misgna et al., (2016) [26]	Tigray	Gulomekada	2016	At least three	Community	Cross-sectional	296	50.0
Fissaha T et al., (2018) [29]	Harar	Harar	2018	At least three	Facility	Cross-sectional	432	32.9
Berhane M et al., (2018) [22]	Oromia	Jimma	2018	At least three	Community	Cross-sectional	422	34.8
Adem N, et al., (2017) [25]	Tigray	Mekelle	2017	At least three	Facility	Cross-sectional	350	50.6
Desalegn et al., (2018) [16]	Amhara	Gondar	2018	At least three	Community	Cross-sectional	845	64.1
Melese et al., (2018) [17]	Amhara	Woldia	2018	At least six	Facility	Cross-sectional	197	11.7
Fekecha B et al., (2017) [19]	SSNPR	Wolkite	2017	At least three	Community	Cross-sectional	368	31.5
Demis B et al., (2018) [28]	AA*	AA*	2018	At least one	Facility	Cross-sectional	512	88.9
Berhea TA et al., (2018) [27]	Tigray	Mekelle	2018	At least three	Community	Cross-sectional	456	66.2
Bulto et al., (2019) [24]	Oromia	Ambo	2019	At least three	Facility	Cross-sectional	404	20.3
Degefa et al., (2019) [21]	**SNNPR	Arba Minch	2019	At least two	Facility	Cross-sectional	345	40.9

*SNNPR Southern nation nationalities and peoples reperesentatives, AA* Addis Ababa

### Women’s knowledge towards neonatal danger signsin Ethiopia

**Fig. 2:**
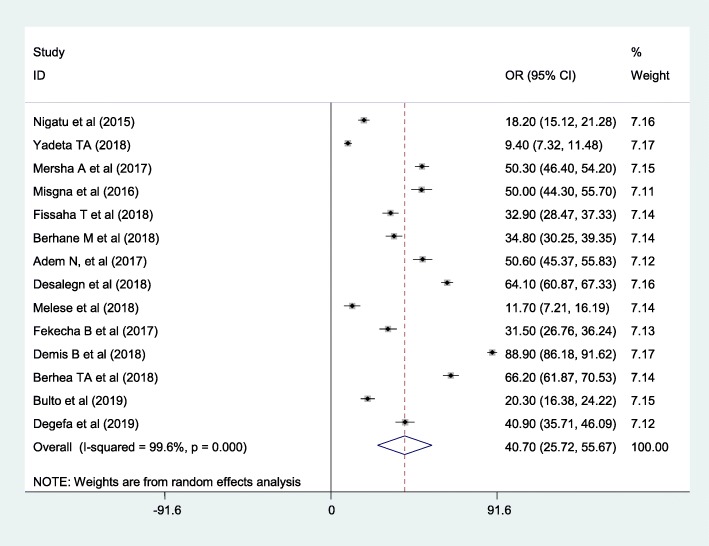
Forest plot of the pooled prevalence of women’s knowledge towards newborn danger signs in Ethiopia

The overall pooled prevalence of mothers knowledge towards newborn danger signs was 40.7% (95%CI, 25.72, 55.67) **(**Fig. 2). High heterogeneity was observed across the included studies (I^2^ = 99.6, *P* < 0.001). As a result, a random-effects model was employed to estimate the pooled prevalence of knowledge of neonatal danger signs in Ethiopia.

### Heterogeneity and publication bias

**Fig. 3:**
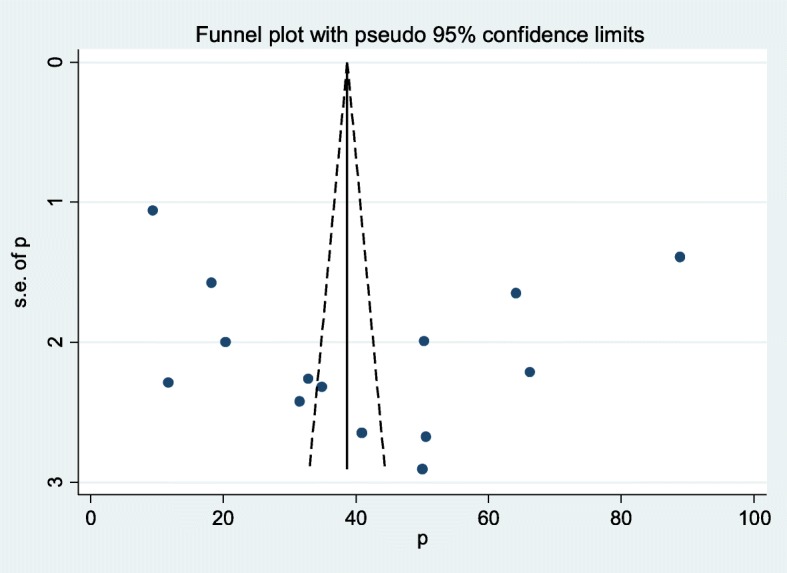
Funnel plot with 95% confidence limits of the pooled prevalence of women’s knowledge towards neonatal danger signs in Ethiopia

The existence of heterogeneity and publication bias was determined within the included studies, as a result, there was considerable heterogeneity across included studies in this meta-analysis (I^2^ = 99.6%). Publication bias was assessed using Egger’s tests, showing no statistically significant for estimating the prevalence of maternal knowledge towards newborn danger signs in Ethiopia (*P* = 0.562). There is symmetrical distribution of included studies in funnel plot which suggests there is no evidence of publication bias (Fig. 3).

### Subgroup analysis

We performed a subgroup analysis based on the study setting and sample size. Therefore after conducting the subgroup analysis of study setting, the pooled prevalence reported in facility based was 40.9% (95% CI: 13.16, 68.59) and community based with a prevalence of 40.6% (95%CI: 23.47, 57.64). Regarding sample size the prevalence of maternal knowledge towards neonatal danger sign was higher in studies with a sample size of > 400 having a prevalence of 42.8% (95%CI: 22.21, 63.28) than studies conducted with a sample size of ≤400 having a prevalence of 36.9% (95%CI, 22.13, 51.62).

Besides, subgroup analysis of women’s knowledge about neonatal danger signs was conducted based on the number of spontaneous responses given by women. Ten articles assessed women’s knowledge towards neonatal danger signs based on at least three spontaneous responses given by women [16, 18–22, 24–27, 29] and the remaining four articles assessed based on at least one spontaneous responses, at least two spontaneous responses, at least four spontaneous responses and at least six spontaneous responses [17, 21, 23, 28].

Accordingly, the level of women’s knowledge about neonatal danger signs with seven individual study populations assessed with at least three spontaneous responses was found to range between 18.2 and 64.4%, with an overall summarized random effect meta-analysis knowledge of 41.9% [95% CI; (30.17, 53.6%), I^2^ = 98.8, *p* < 0.001]. Women’s knowledge about neonatal danger signs with four individual study populations assessed with at least one, two, four and six responses was found to range between 9.4 and 88.9% (Table 2).

**Table 2:** Subgroup prevalence of women’s knowledge towards neonatal danger signs in Ethiopia

Variables	Characteristics	Included studies	Number of Study participants	Prevalence with (95% CI)	I^2^, *P*-value
Study setting	Community based	8	4377	40.6 (23.47, 57.64)	99.5, < 0.001
	Facility based	6	2240	40.9 (13.16, 68.59)	99.6, < 0.001
Sample size	> 400	9	2071	42.8 (22.21, 63.28)	99.7, < 0.001
	< 400	5	4546	36.9 (22.13, 51.62)	97.7, < 0.001
Number of women’s response	At least one	1	512	88.9 (86.14–91.59)	–
	At least two	1	345	40.9 (35.71–46.09)	–
	At least three	10	4806	41.9 (30.16–53.39)	98.8, < 0.001
	At least four	1	757	9.4 (7.32–11.48)	–
	At least six	1	197	11.7 (7.21–16.19)	–
**Overall**		14	6617	40.7 (25.72, 55.68)	99.6, < 0.001

### Factors associated with women’s knowledge towards neonatal danger signs in Ethiopia

#### The association between the educational status of the mother and knowledge of mother towards neonatal danger signs in Ethiopia

**Fig. 4:**
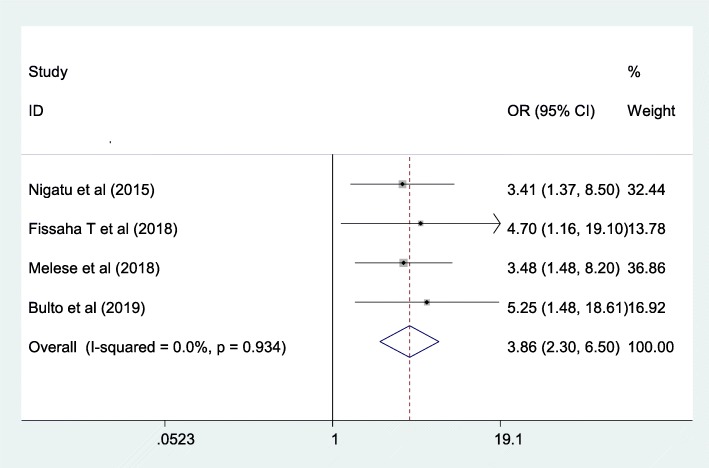
The overall pooled odds ratio of the association between maternal educational status and maternal knowledge on neonatal danger signs level in Ethiopia

Three cross-sectional studies were included to see the association between level of education and knowledge of mothers towards neonatal danger signs. The pooled odds ratio of higher maternal education level were 3.86 times more likely knowledgeable towards neonatal danger signs than their counterparts (AOR = 3.86; 95% CI; 2.3–6.5). These studies hadn’t indicated heterogeneity (I^2^ = 0, *p* = 0.934) with no evidence of publication bias using egger test with *p*-value of 0.076 (Fig. 4).

#### The association between the educational status of the husband and knowledge of mother towards neonatal danger signs in Ethiopia

**Fig. 5:**
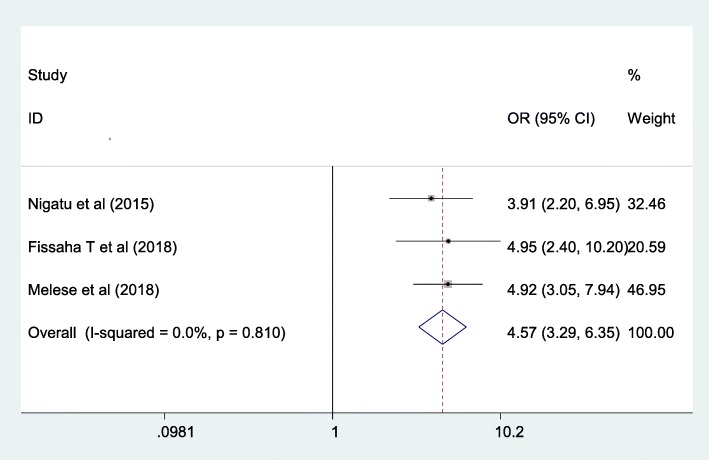
The overall pooled odds ratio of the association between the educational status of the husband and maternal knowledge on neonatal danger signs in Ethiopia

In this meta-analysis, having a higher educational level is 4.57 times more likely to have good knowledge of the mother regarding the danger sign of the newborn. The heterogeneity was not detected in this included studies (I^2^ = 0.0%, *p* = 0.81) without evidence of publication bias using egger test with *p*-value of 0.941 (Fig. 5).

#### Antenatal care follow-up is one of the associated factors for women to have good knowledge towards danger sign of the newborn

**Fig. 6:**
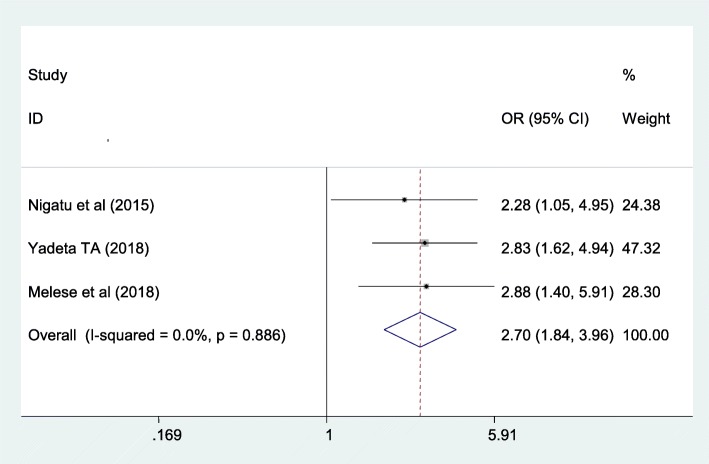
The overall pooled odds ratio of the association between antenatal care follow up and maternal knowledge on neonatal danger signs in Ethiopia

In this study women who had at least one antenatal care follow up were 2.7 times more likely to have good knowledge on danger sign of the newborn than women who hadn’t antenatal care follow up. Heterogeneity was not seen in these meta-analysis of included studies (I^2^ = 0.0%, *p* = 0.886). Possibility of publication bias was seen using egger test with *p*-value of 0.296 (Fig. 6).

#### The association between postnatal care follow-up and knowledge of the mother on danger sign of the newborn in Ethiopia

**Fig. 7:**
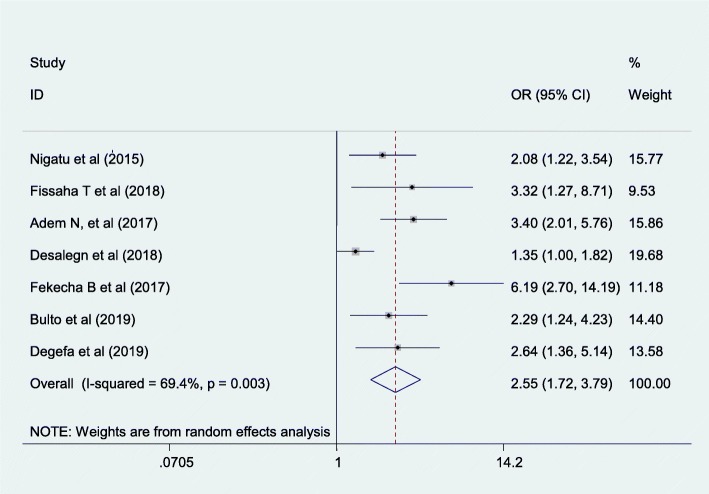
The overall pooled odds ratio of the association between postnatal care follow up and maternal knowledge on neonatal danger signs in Ethiopia

From five cross-sectional studies having postnatal care follow-up was an associated factor for knowledge of the mother on danger sign of the newborn. Having postnatal care follow up were 2.55 more likely to have good knowledge of the mother towards danger sign of the newborn than mothers who hadn’t postnatal care follow-up (AOR = 2.55; 95%CI;1.72–3.79).. In this meta-analysis, the included studies were characterized by moderate heterogeneity (I^2^ = 69.4%; *p* = 0.003) resulting in the use of a random effect meta-analysis model. Publication bias was detected using Egger’s tests with *p*-values of 0.011(Fig. 7).

#### Accessing mass media is one of the associated factors for women to have good knowledge of danger sign of the newborn

**Fig. 8:**
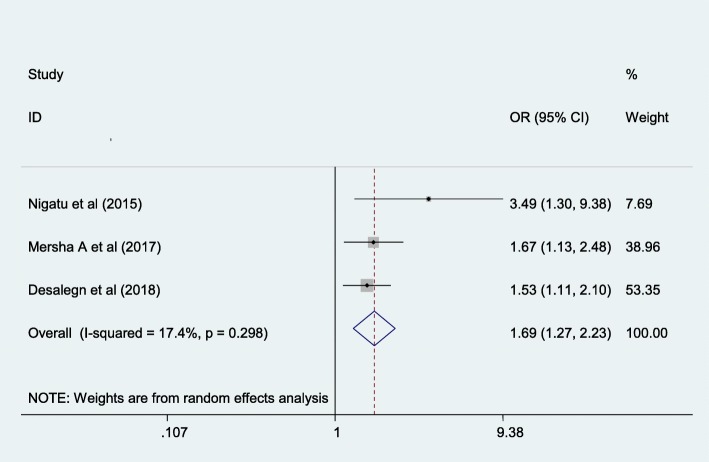
The overall pooled odds ratio of the association between accessing mass media and maternal knowledge on neonatal danger signs in Ethiopia

In this study women who had can access mass media were 1.69 times more likely to have good knowledge of danger sign of the newborn than their counterparts. Minimal heterogeneity was detected in the included studies (I^2^ = 17.4%, *p* = 0.298). Possibility of publication bias was computed using egger test with p-value of 0.296 (Fig. 8).

#### Association between place of delivery and maternal knowledge on danger sign of newborn

**Fig. 9:**
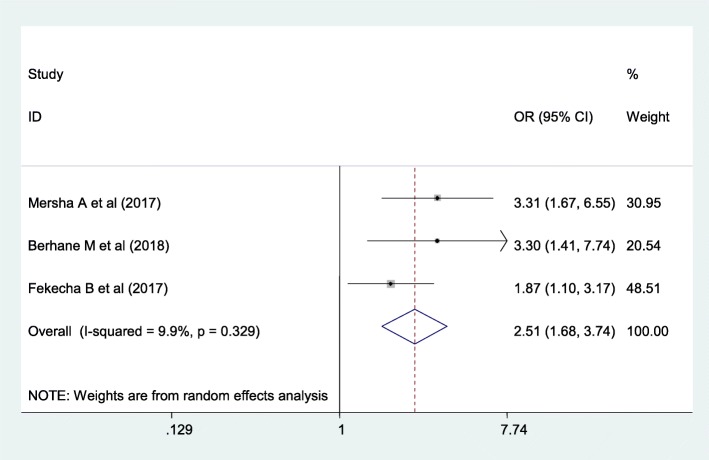
The overall pooled odds ratio of the association between place of delivery and maternal knowledge on neonatal danger signs in Ethiopia

Lastly, meta-analysis was done to see the association between place of delivery and knowledge of the mother on the danger sign of the newborn. Women who gave birth at health institution were 2.51 times more likely to have good knowledge of danger sign of the newborn. The included studies exhibited minimal heterogeneity (I2 = 9.9%, *p* = 0.329) as a result random effect model meta-analysis was used. Publication bias was detected using egger test with a p- value of 1.00 (Fig. 9).

## Discussion

Inadequate knowledge of parents on neonatal danger signs during the neonatal period may perhaps escort to parents’ confusion and decreased quality of care which intimidates the neonatal health and could yet lead to neonatal morbidity and mortality. Therefore, this systematic review and meta-analysis aimed to estimate the pooled prevalence of maternal knowledge towards neonatal danger signs and its associated factors in Ethiopia. In this review, the overall pooled prevalence rate of women’s knowledge on neonatal danger sign was 40.7% (95%CI = 25.72, 55.68. The finding of the study is higher than the study done in Malawi [30], Afghanistan [31] and Ghana [32]. This might be due to variation in time, measurement of newborn danger signs and socio-demographic characteristics of the study population.

The odds of having knowledge on neonatal danger sign were 3.86 times more likely among women having higher educational level than their counterparts. This finding is supported by the studies conducted in Ghana [33], Bangladesh [34], Uganda [35] and Tanzania [36]. This might be justified by an increased chance of the mother’s exposure to postnatal counselling which would possibly increase knowledge of the mother regarding neonatal danger signs.

Having higher educational level of the husband/partner was 4.57 times more likely to understand the neonatal danger signs than their counterparts. This might be due to the fact that an educated husband might positively influence mothers’ knowledge on neonatal danger signs since the husband is the head of the housing member with high decision-making ability.

The odds of having knowledge on neonatal danger sign were 2.7times more likely among antenatal care attended women than those who have no antenatal care follow-up. This finding supported by the studies conducted in Ghana [33], Bangladesh [34]. This might be due to the fact that having ANC visits during pregnancy may have the high chance of getting counselling on maternal and newborn danger signs from healthcare professionals in charge of the service provided which ultimately improves their knowledge on newborn danger signs.

The odds of having knowledge on neonatal danger sign were 2.55 times more likely among postnatal care attended women than those who have no postnatal care follow-up. This finding is supported by the studies conducted in Ghana [33] and Southern Ethiopia [37]. This might be due to the fact that mothers who attended postnatal follow-up have a high chance of getting information from health professionals regarding knowledge of newborn care.

Having access to mass-media was also found to be significantly associated with being knowledgeable with newborn danger signs. Women who had access to mass media were 1.69 times more likely knowledgeable on newborn danger signs as compared with their counterparts. This might be due to mothers who have access to mass media have a high possibility of gaining information regarding newborn danger signs which contribute to shaping one’s mind [38]. Moreover, giving birth at health institutions was found to be another determinant factor of mother’s knowledge about newborn dangers signs. Women who gave birth at health institutions were 2.51 times more likely to have good knowledge k on newborn danger signs as compared to women who gave birth at home. Women who had postnatal care follow up have been counselled about neonatal danger sign and its consequences; hence increasing the knowledge of the women about danger sign of the newborn, might understand the prevention and complications of neonatal problems. Therefore, they become active to recognize early about the type of danger signs of newborns which ultimately increases their knowledge on danger signs of the newborn.

## Conclusion

In this study, maternal knowledge towards neonatal danger signs in Ethiopia was low. Higher educational status of the mother, higher educational status of the husband, access to mass media, having antenatal care follow-up, having postnatal care follow-up, and giving birth at health institutions were factors associated with knowledge of the mother towards danger sign of the newborn. Therefore, based on the study findings, authors recommended that encouraging mother’s to have antenatal care follow-up, postnatal care follow-up and promoting institutional delivery which ultimately increases the potential of mothers to acquire knowledge towards neonatal danger signs.

### Limitation and strength of the study

As strength of the study, we used broader inclusion criteria to include studies conducted both at health facilities and in the community to incorporate a wider range of mother’s knowledge towards neonatal danger signs.

In this study, all the included articles were a study conducted with a cross-sectional design. Therefore, this review shows the level of mother’s knowledge towards neonatal danger signs only at a single point in time, and it is impossible to infer causal relationships among variables. There was no standardized method of measuring thelevel of mother’s knowledge towards neonatal danger signs and hence researchers have used their measuring method. Therefore, the result of this analysis was presented for articles which assessed the level of mother’s knowledge using ‘at least one and above spontaneous responses’ and ‘above mean responses’ making it difficult to pool the level of knowledge together. Furthermore, sub group analysis was employed to see the level of women’s knowledge towards neonatal danger signs.

## Data Availability

All related data has been presented within the manuscript. The dataset supporting the conclusions of this article is available from the authors on request.

## Abbreviations

ANC: Antenatal Care
AA: Addis Ababa
CI: Confidence Interval
CSA: Central Statistical Agency
SNNPR: Southern Nation Nationalities and Peoples Region
UNICEF: United Nation Children’s Emergency Fund
